# Involvement of inflammation in pathogenesis of autism spectrum disorders: experimental model of postnatal valproic acid administration

**DOI:** 10.1192/j.eurpsy.2025.1703

**Published:** 2025-08-26

**Authors:** A. Stakhanova, O. Voskresenskaya, S. Zozulya, N. Kost

**Affiliations:** 1 FSBSI “Mental Health Research Centre”; 2 Lomonosov Moscow State University, Moscow, Russian Federation

## Abstract

**Introduction:**

Autism spectrum disorders (ASD) are developmental deviations that cause social, communication and behavioral problems. Valproic acid (VPA) works as an inhibitor of histone deacetylases. One of the presumed causes of ASD development is neuroinflammation. A low-grade inflammatory process in nervous tissue can cause serious disorders, leading to the death of neurons, impaired myelination and anatomical and biochemical changes in brain structures. Currently, there are known inflammatory markers that are detected in the blood and used to assess the level of inflammation in the brain, for example, leukocyte elastase.

**Objectives:**

To investigate the effect of postnatal administration of VPA on the behavioral manifestations of ASD and the activity of leukocyte elastase in blood serum in male white rats.

**Methods:**

The study was performed on male rat pups. The conditions for keeping animals and the experimental procedures used were approved by the Bioethics Commission of Moscow State University (registration No. 12.3 of 17.11.2022). The animals were administrated intraperitoneally with water (H2O) or VPA at a dose of 150 mg/kg from the 6th to the 12th day of life. The study used standard behavioral tests: weight monitoring from 6-12 days of life, “Social behavior” in the sibling/stranger modification on 55 day. The animals were decapitated on the 57th day. Blood was collected into tubes with serum separating gel (BD Vacutainer® SST™II Advance), centrifuged at 2000 g, serum was frozen -78°C. The activity of leukocyte elastase in blood serum was measured spectrophotometrically by the rate of hydrolysis of the substrate BOC-Ala- ONp (Biomedical, Inc.).

**Results:**

An important physiological indicator for ASD in the model used is a slowdown in weight gain. As a result of the study, it was revealed that the weight of males after 6 days of VPA administration was lower compared to control individuals (p = 0.003). In the “Social Behavior” test, in males who received VPA, the latent period for leaving the starting compartment was significantly longer than in the control (p = 0.019); also, experienced males later approached a stranger as if they were a stranger (p = 0.037), compared to controls. In this study, the activity of leukocyte elastase in the rat’s blood serum receiving VPA was significantly higher than in control individuals (p = 0.04). Similar deviations are observed in patients with ASD.

**Conclusions:**

These results allow us to conclude that the administration of valproic acid to rats in the early postnatal period causes changes in physiological and behavioral characteristic typical for ASD. This confirms the validity of the created experimental model. The increase of leukocyte elastase activity in the rat receiving VPA indicate the role of inflammation in pathogenesis of ASD.

**Disclosure of Interest:**

None Declared

